# Pure Well-Differentiated Adenocarcinoma Is a Safe Factor for Lymph Node Metastasis in T1 and T2 Colorectal Cancer: A Pilot Study

**DOI:** 10.1155/2018/8798405

**Published:** 2018-11-18

**Authors:** Naohisa Yoshida, Masayoshi Nakanishi, Ken Inoue, Ritsu Yasuda, Ryohei Hirose, Yuji Naito, Yoshito Itoh, Tomohiro Arita, Yasutoshi Murayama, Yoshiaki Kuriu, Eigo Otsuji, Akio Yanagisawa, Kiyoshi Ogiso, Takaaki Murakami, Yukiko Morinaga, Eiichi Konishi, Yutaka Inada, Mitsuo Kishimoto

**Affiliations:** ^1^Department of Molecular Gastroenterology and Hepatology, Kyoto Prefectural University of Medicine, Graduate School of Medical Science, Kyoto, Japan; ^2^Department of Surgery, Division of Digestive Surgery, Kyoto Prefectural University of Medicine, Kyoto, Japan; ^3^Department of Pathology, Kyoto First Red Cross Hospital, Kyoto, Japan; ^4^Department of Gastroenterology, JR Osaka railway Hospital, Osaka, Japan; ^5^Department of Gastroenterology, JCHO Kyoto Kuramaguchi Medical Center, Kyoto, Japan; ^6^Department of Surgical Pathology, Kyoto Prefectural University of Medicine, Graduate School of Medical Science, Kyoto, Japan; ^7^Department of Gastroenterology, Fukuchiyama City Hospital, Kyoto, Japan

## Abstract

**Background and Aims:**

Various risk factors for lymph node metastasis (LNM) have been reported in colorectal T1 cancers. However, the factors available are insufficient for predicting LNM. We therefore investigated the utility of the new histological factor “pure well-differentiated adenocarcinoma” (PWDA) as a safe factor for predicting LNM in T1 and T2 cancers.

**Materials and Methods:**

We reviewed 115 T2 cancers and 202 T1 cancers in patients who underwent surgical resection in our center. We investigated the rates of LNM among various clinicopathological factors, including PWDA. PWDA was defined as a lesion comprising only well-differentiated adenocarcinoma. The consistency of the diagnosis of PWDA was evaluated among two pathologists. In addition, 72 T1 cancers with LNM from 8 related hospitals over 10 years (2008–2017) were also analyzed.

**Results:**

The rates of LNM and PWDA were 23.5% and 20.0%, respectively, in T2 cancers. Significant differences were noted between patients with and without LNM regarding lymphatic invasion (81.5% vs. 36.4%, *p* < 0.001), poor histology (51.9% vs. 19.3%, *p* = 0.008), and PWDA (3.7% vs. 25.0%, *p* = 0.015). The rates of LNM and PWDA were 8.4% and 36.1%, respectively, in T1 cancers. Regarding the 73 PWDA cases and 129 non-PWDA cases, the rates of LNM were 0.0% and 13.2%, respectively (*p* < 0.001). Among the 97 cases with lymphatic or venous invasion, the rates of LNM in 29 PWDA cases and 68 non-PWDA were 0% and 14.7%, respectively (*p* = 0.029). The agreement of the two pathologists for the diagnosis of PWDA was acceptable (kappa value > 0.5). A multicenter review showed no cases of PWDA among 72 T1 cancers with LNM.

**Conclusions:**

PWDA is considered to be a safe factor for LNM in T1 cancer.

## 1. Introduction

The incidence of colorectal cancer is increasing worldwide, including in Europe, the USA, and Japan [1]. T2 cancers are generally treated with surgery and show a good prognosis. However, 17–20% of T2 cancers have lymph node metastasis (LNM), and some T2 cancers develop recurrence after operation [2, 3].

While no analyses of risk factors for LNM in colorectal T2 cancers have been reported, there are many reports of the risk factors associated with LNM in T1 cancers. The rate of LNM in T1 cancers is about 10%, which is lower than that of T2 cancers [4–8]. Most T1 cancers are treated surgically because of the risk of LNM. However, these surgeries sometimes cause complications, such as infection, anastomosis leakage, frequent defecation, and sexual-urinary dysfunction. Permanent or temporary artificial anuses are sometimes created for surgery of the lower rectum. Recently, technological improvements have been achieved for endoscopic resection such as endoscopic mucosal resection and endoscopic submucosal dissection, and some T1 cancers can now be resected with free margins [9–11]. It is therefore important for physicians diagnosing T1 cancers to identify cases that can be completely cured by endoscopic resection alone.

Based on the findings of studies regarding the risk factors of LNM, the Japanese Colorectal Cancer Treatment Guidelines note that the following histological findings are associated with LNM: “cancer histology: poorly differentiated adenocarcinomas, signet ring cell carcinomas, or mucinous carcinomas,” “submucosal invasion: ≥1000 *μ*m,” “positive lymphatic and venous invasion,” and “budding: grade 2/3” [4–8]. The guidelines advise physicians to consider additional surgical resection if these predictive factors for LNM are present [12]. However, cases positive for these factors that do receive additional surgery only show LNM in about 10% of cases [4–8, 12]. This shows that these risk factors are not sufficient for predicting LNM.

We therefore hypothesized that safe factors for LNM in T2 cancer are useful for predicting cases without LNM in T1 cancer. In addition, safe factors may reduce the rate of unnecessary additional surgery in T1 cancers treated with endoscopic resection. To our knowledge, safe factors for LNM have not been analyzed previously in T1 and T2 cancers.

In the current study, we investigated the utility of the new histological factor pure well-differentiated adenocarcinoma (PWDA) as a safe factor for LNM in T1 and T2 cancers.

## 2. Materials and Methods

We reviewed 115 T2 cancers and 202 T1 cancers in patients who underwent surgical resection accompanied by lymphadenectomy at Kyoto Prefectural University of Medicine between January 2008 and December 2015. The exclusion criteria were as follows: the presence of clear distant metastasis before surgery; the presence of active malignant disease in any other organs, including other T1–T4 colorectal cancers; patients who underwent preoperative chemotherapy and surgery for colorectal cancers; and pedunculated T1 cancers.

Regarding the 115 T2 cancers, the overall clinicopathological factors, including the rates of LNM and PWDA, were analyzed. For the univariate analysis of LNM, the lesions were then divided into cases with and without LNM, and various clinicopathological factors, such as the age, sex, tumor size, lesion location, lymphatic invasion, venous invasion, budding grade 2/3, histology, and PWDA histology, were analyzed. According to the results of the univariate analysis, the multivariate analysis about LNM was performed to the candidate factors including non-PDWA. Thus, the factors showing *p* value <0.10 in the univariate analysis was analyzed for the multivariate analysis. Moreover, the time to recurrence after the operation was analyzed using a Kaplan-Meier analysis among cases with or without LNM and cases with or without PWDA.

Regarding the 202 T1 cancers, the overall clinicopathological factors, including the rates of LNM and PWDA, were analyzed. For the univariate analysis of PWDA, the lesions then were divided into cases with and without PWDA (the PWDA group and non-PWDA group), and various clinicopathological factors, such as the age, sex, tumor size, lesion location, lymphatic invasion, venous invasion, budding grade 2/3, histology, and LNM status, were analyzed. In addition, we analyzed the relationship between PWDA and LNM in the 97 cases with lymphatic and venous invasion. To assess the consistency of the diagnosis of PWDA, we examined the intra- and interobserver agreement between two pathologists (E.K. and M.K.). Each pathologist reviewed specimens after three weeks.

In addition, 72 T1 cancers with LNM collected from 8 related hospitals for these 10 years (2008–2017) were reviewed for various factors, including the presence of PWDA histology. The 8 institutions were as follows: Kyoto Prefectural University of Medicine, Kyoto, Japan; Nishijin Hospital, Kyoto, Japan; Murakami Memorial Hospital, Gifu, Japan; Kyoto City Hospital, Kyoto, Japan; Matsushita Memorial Hospital, Osaka, Japan; Nara City Hospital, Nara, Japan; Kyoto Kujyo Hospital, Kyoto, Japan; Fukuchiyama City Hospital, Kyoto, Japan; and Ayabe City Hospital, Kyoto, Japan. A gastrointestinal pathologist (M.K.) reviewed all histological specimens of the 72 cases to diagnose the existence of PWDA histology and other histological findings.

Finally, a simulation analysis was performed to determine whether or not PWDA as a safe factor could reduce the number of additional surgeries in endoscopically resected T1 cancers. We hypothesized all 202 T1 cases were resected endoscopically and calculated how many of these T1 cancers resected could have instead simply been followed up according to the risk factors of LNM if PWDA had been adopted as a safe factor.

**Figure 1:**
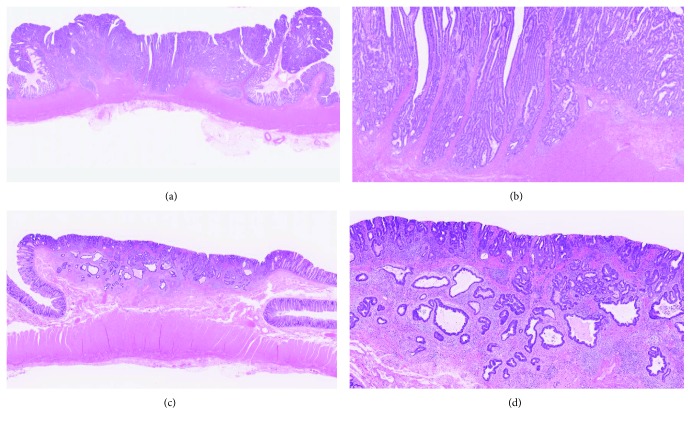
Case presentation of pure well-differentiated adenocarcinoma (PWDA). (a, b) A case of T2 cancer with PWDA. Histology of the surgically resected specimen. The histological diagnosis was T2 cancer constructed only of well-differentiated adenocarcinoma carcinoma. (c, d) A case of T1 cancer with PDWA. Histology of the surgically resected specimen. The histological diagnosis was T1 cancer constructed only of well-differentiated adenocarcinoma carcinoma.

**Figure 2:**
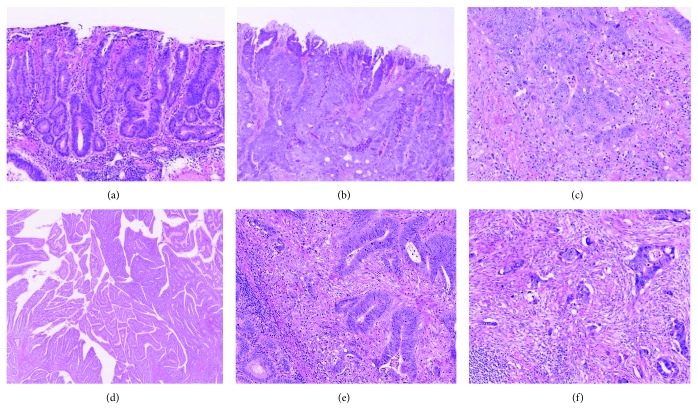
Well-differentiated adenocarcinoma and various other histological findings. (a) Well-differentiated tubular adenocarcinoma. (b) Moderately differentiated tubular adenocarcinoma. (c) Poorly differentiated adenocarcinoma. (d) Papillary differentiated adenocarcinoma. (e) Poor differentiated nest. (f) Budding.

Regarding the histological examinations, all resected specimens were pinned to a flat board and fixed in 10% buffered formalin for 12–48 h. In T2 cancers, the specimens were then cut into 5 mm blocks, while in T1 cancers, the specimens were cut into 2 mm blocks. A PWDA histology was defined as a lesion comprising only well-differentiated tubular adenocarcinoma components in T2 and T1 cancers (Figure 1). Lesions with some moderately differentiated, poorly differentiated or papillary differentiated adenocarcinoma, and poorly differentiated nests or budding in a specimen were diagnosed as non-PWDA (Figure 2). According to the definition of Japanese Classification of Colorectal Carcinoma, well-differentiated tubular adenocarcinoma has a clear, large tubular structure, while moderately differentiated tubular adenocarcinoma has severe structural deformities and ill-formed ducts due to, for example, fusiform ducts or cribriform structures [13]. A poor histology was defined as cancers with poorly differentiated adenocarcinoma in any parts (both partial and main). With respect to budding, Ueno et al. defined budding as “a carcinoma nest composed of a single nest, or else fewer than five component cells that exhibit stromal invasion at the growing front of the tumor.” They classified budding from grades 1 to 3 according to the number of nests visible under a 20x objective and considered grade 2 (5–9 nests) or grade 3 (≥10 nests) to be a risk factor for LNM [14]. On the ohter hand, poorly differentiated nests, which have been reported to be associated with LNM, were defined in previous reports as “carcinoma nests consisting of at least five cells that lack a ductal structure” [15, 16]. Lymphatic and venous invasion was examined using hematoxylin and eosin (HE) staining. Immunohistochemical examinations, such as elastic HE (E-HE) staining for venous invasion and D2-40 for lymphatic invasion, were sometimes performed according to each pathologist's decision. A gastrointestinal pathologist (M.K.) reviewed all histological specimens to confirm the diagnoses of PWDA histology.

This study, including the multicenter review, was conducted with the approval of the Ethics Committee of Kyoto Prefectural University of Medicine (ERB-C-794). In addition, this study was also performed in accordance with World Medical Association Declaration of Helsinki.

### 2.1. Statistical Analyses

Statistical analyses were performed using the SPSS software program (version 22.0 for Windows; IBM Japan Ltd., Tokyo, Japan). Categorical variables were examined using chi-squared tests. Continuous variables, such as the patient age and lesion size, were analyzed using a Mann-Whitney *U* test. Multivariate logistic regression analyses were constructed for the determination of independent risk factors for LNM in T2 cancers. A *p* value <0.05 was considered statistically significant. Additionally, the 95% confidence interval for the LNM rate related with lymphatic or venous invasion in both the PWDA group and non-PWDA group was estimated with a binomial distribution because our sample size was less than 100.

## 3. Results

In the 115 T2 cancers, the rate of lymphatic invasion was 47.0% (54 cases); venous invasion, 48.9% (59 cases); budding grade 2/3, 38.3% (49 cases); and poor histology, 27.0% (31 cases) (Table 1). The overall rates of PWDA and LNM were 20.0% (23 cases) and 23.5% (27 case), respectively. In the 202 T1 cancers, the rate of lymphatic invasion was 33.6% (68 cases); venous invasion, 24.2% (49 cases); budding grade 2/3, 13.4% (27 cases); and poor histology, 12.3% (25 cases). The overall rates of PWDA and LNM were 36.1% (23 cases) and 8.4% (17 cases), respectively.

The results of a comparison of cases with and without LNM among T2 cancers are shown in Table 2. Significant differences were noted between cases with and without LNM in the rates of rectal location (70.4% vs. 42.0%, *p* = 0.013), tumor size ≥25 mm (77.8% vs. 55.7%, *p* = 0.039), lymphatic invasion (81.5% vs. 36.4%, *p* < 0.001), budding grade 2/3 (55.6% vs. 33.0%), poor histology (51.9% vs. 19.3%, *p* = 0.008), and PWDA (3.7% vs. 25.0%, *p* = 0.015). Multivariate analyses showed rectal location was an independent risk factor (odds ratio (OR), 6.848; 95% confidence interval (CI), 1.661–28.239; *p* = 0.008) (Table 3). Tumor ≥25 mm (OR, 9.583, 95% CI, 2.027–45.317; *p* = 0.004), lymphatic invasion (OR, 10.276, 95% CI, 2.181–48.424; *p* = 0.003), and poor histology (OR, 4.773, 95% CI, 1.135–20.073; *p* = 0.033) were also risk factors. On the contrary, non-PWDA was not a significant risk factor (*p* = 0.443).

**Figure 3:**
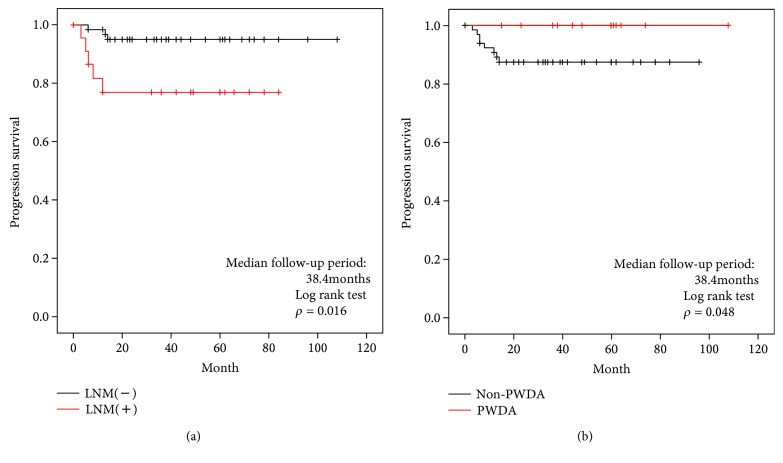
Time to tumor recurrence after operation stratified by LNM and PDWA in T2 cancers. PWDA: pure well-differentiated adenocarcinoma; LNM: lymph node metastasis.

The time to tumor recurrence after operation based on the LNM and PDWA among T2 cancers is shown in Figure 3. A significant difference was noted between cases with and without LNM (*p* = 0.016). Furthermore, cases with PWDA showed a significantly better prognosis than those without PWDA (*p* = 0.048), and there were no deaths in the PWDA group.

The results of a comparison of cases with and without PWDA among T1 cancers are shown in Table 4. Significant differences were noted between cases with and without PDWA in the incidence of colonic location (71.2 vs. 56.7%, *p* = 0.039), ratio of T1a (27.4% vs. 7.8%, *p* < 0.001), submucosal invasion length (2317 ± 1953 *μ*m vs. 3730 ± 2722 *μ*m, *p* < 0.001), and lymphatic invasion (24.7% vs. 38.8%, *p* = 0041). Furthermore, a significant difference in the rate of LNM was noted between cases with and without PWDA (0% vs. 13.2%, *p* < 0.001).

Among the 73 PWDA cases, 44 without lymphatic or venous invasion had no LNM and 29 with lymphatic or venous invasion had no LNM (Table 5). In contrast, among the 129 non-PWDA cases, the 61 without lymphatic or venous invasion had an LNM rate of 11.5% and the 68 with lymphatic or venous invasion had an LNM rate of 14.7%. A significant difference in the LNM was noted between PWDA cases with lymphatic or venous invasion and non-PWDA cases with lymphatic or venous invasion (*p* = 0.029).

With respect to histological agreement concerning PWDA, the kappa values for the intraobserver agreement of the 2 pathologists were 0.612 and 0.681, while that for the inter-observer agreement concerning PWDA was 0.596.

The results of a multicenter review of T1 cancers with LNM in 8 hospitals are shown in Table 6. The rate of lymphatic or venous invasion was 63.9%, and there were no PWDA cases among the 72 T1 cancers with LNM.

**Figure 4:**
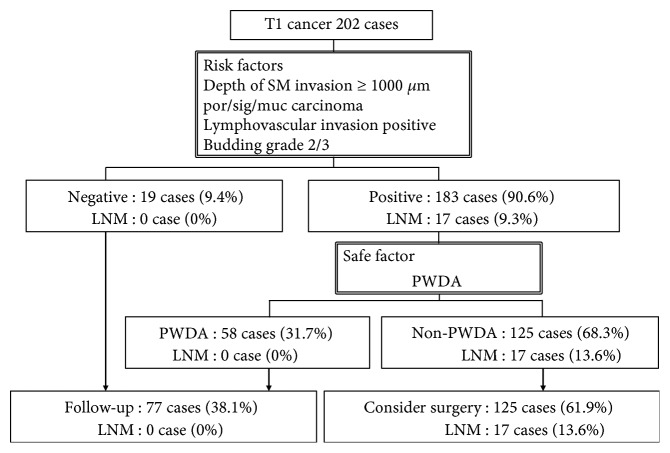
A simulation adopting PDWA as a safe factor in T1 cancers. PWDA: pure well-differentiated adenocarcinoma; SM: submucosal; LNM: lymph node metastasis.

The simulation study showed that 183 of 202 T1 cancers (90.6%) should be considered for surgical operation according to the 4 proposed risk factors (Figure 4). All LNM cases were included among these 183 patients. When PWDA was adopted as a safe factor, none of the 58 patients with PWDA had LNM. A total of 77 of the 202 T1 patients (38.1%) could be followed up without additional surgical operation.

## 4. Discussion

In the current study, we showed that T2 cancer patients with PWDA had a lower rate of LNM (4.3%) than those without PDWA (28.3%) and a better prognosis as well. In T1 cancers, the LNM rate among PDWA cases (0%) was significantly lower than that among non-PWDA cases (13.2%). In addition, no PWDA cases with positive lymphatic or venous invasion had any LNM. A multicenter review also showed that none of the 72 T1 cancer patients with LNM had PWDA. The adoption of PDWA has a possibility of more than 30% of decrease for additional surgery after the endoscopic resection of T1 cancers.

These findings prove that PWDA is a novel histological safe factor for LNM and indicate a better prognosis in T2 cancer patients. PWDA may therefore be useful as an absolute definite safe factor for LNM in T1 cancer. Tanaka et al. reported that well- or moderately well-differentiated lesions showed LNM in 4.9% (19/388) of cases [17]. In contrast, poorly moderately or poorly differentiated lesions showed LNM in 37.3% (25/67) of cases. In addition, well- or moderately well-differentiated lesions within a submucosal invasion length of 1500 *μ*m showed no LNM. They concluded that the combination of these two findings without lymphatic or venous invasion was considered a safe factor. Another report showed that well-differentiated adenocarcinomas with low-grade atypia had a low risk of LNM. The report subclassified well-differentiated adenocarcinoma into carcinoma with high-grade atypia and carcinoma with low-grade atypia based on their cellular atypia [18]. This definition is different from the definition of PDWA because we did not consider the cellular atypia for the diagnosis of PWDA. We especially focused on histological structural deformities for diagnosing PWDA. Thus, only the lesions with tubular well-differentiated adenocarcinoma in whole specimens were diagnosed as PDWA. On the contrary, well-differentiated adenocarcinoma lesions with only a small part of moderately differentiated, poorly differentiated or papillary differentiated adenocarcinoma, and poorly differentiated nests or budding in a specimen were diagnosed as non-PWDA. According to this definition, some of PDWAs might include low-grade atypia though we did not analyze it.

PWDA is deemed an even safer factor than this because it was found to be a safe factor even in cases with lymphatic or venous invasion. In addition, variations in the degree of submucosal invasion sometimes occur since the muscularis mucosa is subjectively determined by each pathologist [4]. As histological risk factors, many previous studies have identified factors listed in the guideline, such as lymphatic and venous invasion, submucosal invasion distance, poor histology, and budding [4–8]. In addition, the presence of cribriform structures, which is a neoplastic epithelial proliferation in the form of large nests perforated by many rounded crypts of different sizes, was reported to be associated with LNM and a low survival rate in moderately differentiated adenocarcinoma [19]. Compared to these risk factors, non-PWDA is not a risk factor for LNM as the multivariate analysis about T2 cancers showed. Actually, non-PDWA includes some cases safe from LNM, as PDWA is used to select absolutely safe cases. We therefore did not perform a multivariate analysis of risk factors including non-PWDA for T1 cancer in this study. We expect that physicians can determine the LNM risk associated with endoscopically resected T1 cancer by performing risk stratification assuming a PWDA histology to be an absolute safe factor for LNM, in combination with other risk factors. However, our simulation study showed that a PWDA histology was able to reduce the need for additional surgery in cases of endoscopically resected T1 cancer, although this analysis was performed among operated cases. Therefore, a further multicenter large-scale study will be required in order to examine the efficacy of PDWA as a safe factor for LNM.

For the diagnosis of PWDA, the kappa values for the intra- and interobserver agreement of the 2 pathologists were not high enough (0.596–0.681) though this study was performed only in one center. This is partially due to the different definition of well-differentiated adenocarcinoma in each pathologist. The consistent diagnosis for well-differentiated adenocarcinoma is expected for increasing the agreement. We consider the reference of some typical images like Figures 1 and 2 probably results in the increase of diagnostic agreement of PDWA. Compared to this, lymphatic and venous invasion and budding also have low diagnostic consistency between pathologists [20]. HE-based assessments are frequently subjective, and various immunohistochemical staining approaches are used to diagnose lymphatic and venous invasion. In Japan, immunohistochemical staining with the monoclonal D2-40 antibody, which reacts with the O-linked sialoglycoprotein (molecular weight 40 kDa) on the lymphatic endothelial surface, enables the differentiation of lymphatic channels and small vessels and is used for evaluating lymphatic invasion in Japan. Similarly, EVG or E-HE is used to stain and identify elastic fibers in the venous walls and to evaluate venous invasion. In the West, ERG staining is sometimes adopted to assess lymphatic and venous invasion. However, the performance of these immunohistochemical examinations is left to the pathologist's decision, and HE staining is generally used globally. Of note, a previous study reported that the degree of interobserver agreement among pathologists for lymphatic and venous invasion is substantial with HE staining (kappa value = 0.18–0.28) and did not improve upon the use of immunohistochemical staining (0.42) [21]. This suggests that PWDA may be more objective than these difficult evaluations of lymphatic and venous invasion. However, the histological agreement of PWDA should be examined in greater detail by more pathologists at several centers, as it was performed only by two pathologists in the same center in our study.

Histological heterogeneity sometimes happens such that various histological subtypes are mixed in routine histological examination. Actually, some of well-differentiated adenocarcinoma have a small part of moderately differentiated adenocarcinoma or poorly differentiated adenocarcinoma especially in the deepest part. According to WHO classification, the histological grade 1 is defined a lesion with more than 95% of well-differentiated adenocarcinoma [22]. Compared to this, PDWA was defined as a lesion with 100% of well-differentiated adenocarcinoma. Thus, we should check whole histological specimens for diagnosing PDWA accurately.

At present, T2 colorectal cancers cannot be resected endoscopically, even if an ESD technique is used. However, recent improvements in endoscopic surgery have showed a potential of part of T2 cancer and full wall resection of the colorectum [23]. Such an analysis of the risk and safe factors for LNM in T2 cancer will be more useful when T2 cancers can be resected and the need for additional surgery after endoscopic resection must be determined.

Several limitations associated with the present study warrant mention. This study had a retrospective design and was conducted by only one pathologist in our center. Furthermore, there were a small number of T2 and T1 cancer cases. In addition, the number of T1 cancer cases with LNM was insufficient to prove PWDA was a safe factor for LNM. Moreover, we did not analyze pedunculated T1 cancers in our study because we did not have enough numbers of those cases with LNM. A further study is expected whether PWDA can be applied to them.

## 5. Conclusions

Our pilot study showed that PWDA is a safe factor for predicting LNM in T1 cancers. PWDA in T1 cancer patients can facilitate risk stratification for LNM, although further large-scale studies should be performed.

## Figures and Tables

**Table 1:** Clinicopathological characteristics of 115 T2 and 202 T1 colorectal cancers.

	T2 cancers	T1 cancers
Case numbers	115	202
Age, years; mean (range)	67.8 ± 10.8 (37–85)	65.8 ± 9.9 (24–88)
Sex (male/female) %, (*n*)	53.9 (62)/46.1 (53)	57.9 (117)/42.1 (85)
Tumor size, mm; mean (range)	29.5 ± 12.8 (12–78)	21.6 ± 12.3 (5–80)
Location (colon/rectum) %, (*n*)	50.4 (58)/49.6 (57)	61.9 (125)/38.1 (77)
Lymphatic invasion, %, (*n*)	47.0 (54)	33.6 (68)
Venous invasion, %, (*n*)	48.9 (59)	24.2 (49)
Budding grade 2/3%, (*n*)	38.3 (44)	13.4 (27)
Poor histology %, (*n*)	27.0 (31)	12.3 (25)
PWDA %, (*n*)	20.0 (23)	36.1 (73)
LNM %, (*n*)	23.5 (27)	8.4 (17)

PWDA: pure well-differentiated adenocarcinoma; LNM: lymph node metastasis.

**Table 2:** The clinicopathological analysis between cases with and without LNM among T2 cancers.

	Cases with LNM	Cases without LNM	*p* value
Case number	27	88	
Age, years; mean (range)	65.2 ± 12.3 (37–83)	68.8 ± 10.3 (37–94)	0.201
Sex (male/female) %, (*n*)	54.5 (14)/45.5 (13)	55.7 (48)/44.3 (40)	0.922
Location (colon/rectum) %, (*n*)	29.6 (8)/70.4 (19)	58.0 (50)/42.0 (38)	0.013
Tumor size, mm; mean(range)	33.9 ± 14.9 (15–78)	28.1 ± 12.0 (12–70)	0.072
Tumor size ≥25 mm %, (*n*)	77.8 (21)	55.7 (49)	0.039
Lymphatic invasion %, (*n*)	81.5 (22)	36.4 (32)	<0.001
Venous invasion %, (*n*)	66.7 (18)	46.6 (41)	0.067
Budding grade 2/3%, (*n*)	55.6 (15)	33.0 (29)	0.034
Poor histology %, (*n*)	51.9 (14)	19.3 (17)	0.008
PWDA %, (*n*)	3.7 (1)	25.0 (22)	0.015

LNM: lymph node metastasis; PWDA: pure well-differentiated adenocarcinoma.

**Table 3:** The multivariate analysis of the risk factors of LNM in T2 cancers.

	Multivariate analysis		
	OR	95% CI	*p* value
Location rectum (vs. colon)	6.848	1.661 , 28.239	0.008
Tumor size ≥25 mm (vs. <25 mm)	9.583	2.027 , 45.317	0.004
Lymphatic invasion	10.276	2.181 , 48.424	0.003
Poor histology	4.773	1.135 , 20.073	0.033
Non-PWDA	1.620	0.032 , 4.511	0.443

LNM: lymph node metastasis: PWDA: pure well-differentiated adenocarcinoma; OR: odds ratio; CI: confidence interval.

**Table 4:** The difference in various clinicopathological factors between cases with and without PWDA among T1 colorectal cancers.

	PWDA	Non-PWDA	*p* value
Case number	73	129	
Age, years; mean (range)	65.7 ± 10.4 (32–84)	66.0 ± 9.7 (24–88)	0.849
Sex (male/female) %, (*n*)	57.5 (42)/42.5 (31)	58.1 (75)/41.9 (54)	0.933
Tumor size, mm; mean (range)	20.7 ± 11.9 (5–56)	22.3 ± 12.6 (5–80)	0.384
Location (colon/rectum) %, (*n*)	71.2 (52)/28.8 (21)	56.7 (73)/43.3 (56)	0.039
Morphology (polypoid/nonpolypoid) %, (*n*)	54.8 (40)/45.2 (33)	65.1 (84)/34.9 (45)	0.147
SM invasion (T1a/T1b) %, (*n*)	27.4 (20)/72.6 (53)	7.8 (10)/92.2 (119)	<0.001
SM invasion distance, *μ*m; mean (range)	2317 ± 1953 (50–10,000)	3730 ± 2722 (100–14,000)	<0.001
Lymphatic invasion %, (*n*)	24.7 (18)	38.8 (50)	0.041
Venous invasion %, (*n*)	21.9 (16)	25.6 (33)	0.559
Budding grade 2/3%, (*n*)	0 (0)	21.0 (27)	<0.001
Poor histology %, (*n*)	0 (0)	19.4 (25)	<0.001
LNM %, (*n*)	0 (0)	13.2 (17)	<0.001

PWDA: pure well-differentiated adenocarcinoma; LNM: lymph node metastasis.

**Table 5:** The relationship of LNM between PWDA and lymphatic or venous invasion in T1 cancers.

	Lymphatic or venous invasion	*N*	LNM % [95% CI], (*n*)
PWDA 73 cases	Negative	44	0.0 [0–8.0] (0)
	Positive	29	0.0 [0–12.0] (0)^∗^
Non-PWDA 129 cases	Negative	61	11.5 [4.7–22.0] [7]
	Positive	68	14.7 [7.3–25.4] [10]^∗∗^

^∗^ vs. ^∗∗^, *p* = 0.029. PWDA: pure well-differentiated adenocarcinoma; LNM: lymph node metastasis; CI: confidence interval.

**Table 6:** A multicenter review analyzing 72 T1 cancers with LNM.

Case number	72
Age, years; mean (range)	64.7 (27–83)
Sex (male/female) %, (*n*)	50.0 (36)/50.0 (36)
Tumor size, mm; mean (range)	21.0 (5–55)
Location (colon/rectum) %, (*n*)	55.6 (40)/44.4 (32)
Morphology (polypoid/nonpolypoid) %, (*n*)	48.6 (35)/51.4 (37)
SM invasion distance, *μ*m; mean (range)	4158.7 (850–11,000)
SM invasion (T1a/T1b) %, (*n*)	2.8 (2)/97.2 (70)
Lymphatic invasion positive %, (*n*)	45.8 (33)
Venous invasion positive %, (*n*)	36.1 (26)
Lymphatic or venous invasion positive %, (*n*)	63.9 (46)
Budding grade 2/3%, (*n*)	22.2 (16)
Poor or mucinous histology %, (*n*)	45.8 (33)
PWDA %, (*n*)	0.0 (0)

LNM: lymph node metastasis; PWDA: pure well-differentiated adenocarcinoma.

## Data Availability

The patient data used to support the findings of this study are available from the corresponding author upon request. However, some of these data are restricted by the institutional review board of the Kyoto Prefectural University of Medicine.
